# Completeness of notifications of severe acute respiratory syndrome at the national level and in a regional health care in the state of Minas Gerais, during the COVID-19 pandemic, 2020

**DOI:** 10.1590/S1679-49742022000200004

**Published:** 2022-06-15

**Authors:** Fábio Vieira Ribas, Ana Cristina Dias Custódio, Luana Vieira Toledo, Bruno David Henriques, Catarina Maria Nogueira de Oliveira Sediyama, Brunnella Alcântara Chagas de Freitas

**Affiliations:** 1 Universidade Federal de Viçosa, Programa de Pós-Graduação em Ciências da Saúde, Viçosa, MG, Brazil; 2 Universidade Federal de Viçosa, Departamento de Medicina e Enfermagem, Viçosa, MG, Brazil

**Keywords:** Severe Acute Respiratory Syndrome, COVID-19, Health Information Systems, Public Health Surveillance

## Abstract

**Objective:**

To analyze the completeness of notifications of severe acute respiratory syndrome cases on the Influenza Epidemiological Surveillance Information System (SIVEP-Gripe) during the COVID-19 pandemic, on the national database and on the Regional Health Center database of the state of Minas Gerais, Brazil, in 2020.

**Methods:**

This was a descriptive study of the completeness of sociodemographic variables and those related to etiology, clinical condition, evolution and diagnostic criteria of SIVEP-Gripe. The level of completeness was classified as excellent (> 95%), good (90% to 95%), regular (80% to 90%), poor (50% to 80%) or very poor (< 50%).

**Results:**

The percentage of variables with excellent completeness was only 18.1% on the national database, and 27.8% on the regional database.

**Conclusion:**

Both SIVEP-Gripe databases presented low completeness, making it necessary to improve the work process and routine training of professionals aimed at the correct filling.

**Study contributions**

**Main results**

Completeness of notifications of hospitalized SARS cases on SIVEP-Gripe at two distinct levels, regional and national, was low. At the national level, there were fewer variables with a level of completeness classified as 'excellent' or 'good'.

**Implications for services**

The low completeness of data compromises the analysis of the epidemiological profile of cases and makes it difficult the management actions of health services.

**Perspectives**

The results can support professionals and managers in order for them to improve the work process and filling the SIVEP-Gripe data, especially regarding variables whose completeness was classified as 'poor' or 'very poor'.

## INTRODUCTION

Severe acute respiratory syndrome (SARS) is considered a flu-like syndrome complication that requires hospital treatment. It is characterized by the simultaneous presence of a flu-like syndrome picture, dyspnea and/or signs of severity, such as oxygen saturation (SpO_2_) < 95% in ambient air, respiratory distress and lips or face with a bluish color.^1^ ^,^ ^2^

This syndrome became, once again, a worldwide concern in 2020, when the World Health Organization (WHO) declared the outbreak of a novel coronavirus, SARS-CoV-2, identified in Wuhan (China) in December 2019.^3^ ^,^ ^4^ The disease caused by SARS-CoV-2 infection, named COVID-19, was declared a public health emergency due to the high morbidity and mortality it had caused.^5^ Individuals with COVID-19 may be asymptomatic, present with mild signs and symptoms of flu-like syndrome, or even moderate, severe and critical conditions, such as SARS cases. Thus, epidemiological surveillance of SARS stood out again, given the importance of monitoring this disease.^6^

For an adequate and systematic monitoring of COVID-19 cases, it is necessary to use health information systems (HIS). HIS has a complex organization, involving the stages of data collection and processing, production and transmission of information, serving as a support for health services management.^4^ That is, the analysis of information obtained from these systems influences the work process of health professionals, daily life of the population, guides managers during the decision-making process and helps to develop health policies.^7^ ^,^ ^8^

In Brazil, Influenza Epidemiological Surveillance Information System (SIVEP-Gripe) is the HIS for the record of universal (and immediate) notification of SARS cases, responsible for recording the data of sentinel surveillance of flu-like syndrome cases, in addition to all hospitalized cases and deaths due to SARS. SIVEP-Gripe was developed to support influenza surveillance actions, mainly for the identification of the main respiratory viruses circulating in the national territory.^6^ ^,^ ^7^ ^,^ ^9^

Due to the severity of the clinical picture and the risk of worsening, SARS cases require hospital beds. Consequently, the notification on SIVEP-Gripe is made in a hospital setting.^1^ ^,^ ^2^ In 2019, Brazil had 8,139 hospital establishments and 490,397 beds, an offer equivalent to approximately 2.3 beds per 1,000 inhabitants.^10^ The Southeast macro-region of Minas Gerais, in 2020, had 51 hospital establishments (including private and public) and 4,743 beds available, or an offer of 2.8 beds per 1,000 inhabitants in that region.^11^ Disaggregating these data at the region level, the Regional Health Center of Ubá (RHC/Ubá), an administrative division of health services in the state of Minas Gerais and the object of this study, had 1,185 hospital beds available, an offer of 2.4 beds per 1,000 inhabitants, an index similar to that of the average national supply in the same year.^11^ These data show the critical situation of the health system to meet the potential demand generated by the COVID-19 pandemic, taking into consideration that, in a non-pandemic situation, the WHO recommends an average of 3 to 5 beds per 1,000 inhabitants.^11^

The challenge that the COVID-19 pandemic represents for the provision of health care quality, safety and evidence-based, requires reliability of SARS notifications on SIVEP-Gripe, by managers, which is important in order to support the public health decision-making.^8^ ^,^ ^12^ In order to ensure this reliability, it is crucial that the records of SARS cases be complete, updated and reliable, especially in the context of the COVID-19 pandemic.^8^ ^,^ ^11^

Taking into consideration the heterogeneity regarding the completeness of the SIVEP-Gripe records among the five Brazilian regions and that, incomplete notifications can negatively impact health surveillance actions, it is essential to conduct studies on the subject. However, studies that evaluate the completeness of data from the SARS surveillance system in Brazil are scarce.

In this context, this work aimed to analyze the completeness of notifications of SARS cases on the SIVEP-Gripe during the COVID-19 pandemic, on the national database and on a regional health database in the state of Minas Gerais, in 2020. The evaluation of both scenarios will allow the analysis of the completeness of this system at the local and national contexts, enabling future interventions aimed to improve the quality of the data produced.

## Methods

This was a descriptive study on data available in SIVEP-Gripe. We analyzed and compared the completeness of the information of hospitalized SARS cases among those living in the municipalities belonging to RHC/Ubá (regional data) and cases of SARS reported throughout the national territory (national data).

The RHC/Ubá includes the health micro-region of Muriaé, with 11 municipalities and a population of 173,744 inhabitants, and the health microregion of Ubá, comprised of 20 municipalities and a population of 314,647 inhabitants.^10^ The RHC/Ubá was selected because it was located in the region where the researchers involved in this study conduct their activities.

We analyzed the cases of SARS who were hospitalized and reported on SIVEP-Gripe between epidemiological weeks 1 and 53 of 2020. Access to the regional SIVEP-Gripe database was possible with a prior authorization of the RHC/Ubá manager. The regional database was used in order to obtain data free of duplicates, given that they had the nominal records, thus it would be possible to exclude the existing duplicates, before analyzing the data. National data, in turn, were obtained from the public access non-nominal database, available at the following website: https://opendatasus.saude.gov.br, on April 26, 2021.

SIVEP-Gripe notification form includes data related to sociodemographic, clinical and diagnostic investigation characterization of cases, whose filling in the system can be internal, essential or mandatory.^13^ During the year 2020, four updates were made, in which the filling rules were modified, including or excluding fields. Therefore, in the evaluation of completeness, the variables classified as essential were selected, available in the system, excluding mandatory filling variables, whose absence of filling constitutes an impeding factor to the registration of the notification form in the system. However, the variable 'self-declared race/skin color' was maintained in the analysis, as it became a mandatory filling variable after the form was updated, on July 27, 2020. In order to avoid errors in the analysis, variables that were suppressed during update of the forms, were also excluded.

Finally, 83 variables were included and grouped into three blocks:

- Sociodemographic and etiological variables: self-declared race/skin color, schooling level, Zip Code of the place of residence, geographic area of the residential address, case arising from flu-like syndrome outbreak, case of SARS with hospital-acquired infection and case with direct contact with birds, pigs, others.
- Clinical condition and evolution: i) signs and symptoms (fever, cough, sore throat, dyspnea, respiratory distress, saturation < 95%, diarrhea, vomiting, abdominal pain, fatigue, loss of smell, loss of taste, other signs and symptoms); ii) risk factors: the individual presents a risk factor, being a puerperal woman, chronic cardiovascular disease, chronic hematologic disease, Down syndrome, chronic liver disease, asthma, diabetes *mellitus*, neurological disorders, chronic lung disease, immunodeficiency or immunodepression, chronic kidney disease, obesity, body mass index (BMI) value (obese people), other risk factors, and descriptions of other morbidities; iii) vaccination history: got vaccinated against influenza in the last campaign, date of his/her last dose of influenza vaccine, mother got vaccinated against influenza (those under 6 months old), mother breastfeeds her child (those under 6 months old); iv) other clinical information: used antiviral medications to treat the disease, antiviral medications used (oseltamivir, zanamivir, other), description of the other antiviral medication used, date of antiviral treatment initiation, need for hospitalization, date of hospitalization, Federative Unit where the hospitalization occurred, municipality where the hospitalization occurred, sentinel unit where the hospitalization occurred, hospitalization in intensive care unit (ICU), date of admission to the ICU, date of discharge from the ICU, use of ventilatory support, evolution of the case, date of discharge or death, date of case closure.
- Diagnostic criteria: i) imaging tests (chest X-ray, specification of other chest X-ray results, date of chest X-ray), tomography result (date of CT scan procedure); ii) sample collection (sample collection was performed for diagnostic test, date of sample collection for diagnostic test, type of clinical sample collected for diagnostic test), specification of another sample that was collected; iii) antigen rapid test (type of antigen rapid test, date of antigen rapid test result, antigen rapid test result, positive antigen rapid test result for influenza); iv) RT-PCR [reverse transcription polymerase chain reaction (RT-PCR) result/another method applying molecular biology, date of RT-PCR test result/another method applying molecular biology, positive RT-PCR test result for influenza, positive RT-PCR result for influenza A or B]; v) serological test (type of serological sample that was collected, description of the type of clinical sample different from those aforementioned, date of collection of the material for serological diagnosis, type of serological test that was performed, description of the type of serological test that was performed, serology result for SARS-CoV-2 IgG, IgM and IgA); vi) outcome (final classification of the case, indicate the confirmation criterion).

Data were analyzed using the IBM SPSS Statistics software, version 23. Absolute and percentage frequency distribution of completeness of variables was described. For the calculation of the completeness of each variable, the set of notifications eligible for its filling was considered as denominator, given that the filling of some variables depends on the answer option of the previous question, considered as a funnel variable, for example: *Do you have any risk/comorbidity factors?*; *Did you get the flu vaccine in the last campaign?*; *Did you use any antiviral medications for influenza?*; *Was there hospitalization?*; *Did you perform a sample collection?*; Final classification of the case; and evolution of the case.

The level of completeness of the studied variables was defined according to the following classification: excellent (completeness > 95%); good (90% to 95% completeness); regular (80% to 90% completeness); poor (50% to 80% completeness); very poor (completeness < 50%).^14^ The fields filled out on the SIVEP-Gripe corresponding to the category 'ignored', zero number, ignored date or lack of information were considered incomplete.^15^

The study project was developed in accordance with the ethical principles set out in the National Health Council, Resolution No. 466, of December 12, 2012, and submitted to the Research Ethics Committee of the institution. It was approved, opinion No. 4,231,826 (Certificate of Submission for Ethical Appreciation No. 36607820.4.0000.5153), on August 24, 2020.

## Results

A total of 1,192,518 cases of SARS were reported on the SIVEP-Gripe system throughout the national territory in 2020, and of these, 2,590 lived in the municipalities belonging to the RHC/Ubá, representing 0.22% of the total. The number of variables with completeness greater than 95%, classified as excellent, was 15 at the national level and 23 at the regional level.

Table 1 presents the completeness of the variables related to the sociodemographic and etiological characterization of reported SARS cases, at national and local levels. Of the seven variables analyzed, 'excellent' completeness was found in three on the regional database, and only one on the national database. The percentage of completeness of the variable 'schooling level' was 33.9% and 37.2% on the regional and national databases, respectively, being classified as 'very poor' level of completeness.

**Table 1:** Completeness of the blocks of variables related to sociodemographic and etiological characterization of hospitalized SARS^a^ cases (n = 1,192,518), at the regional and national levels, according to the SIVEP-Gripe,^b^ for the Regional Center of Ubá/MG and Brazil, 2020

Variables	Completeness	
	Regional^c^ (%)	National^d^ (%)
1. Race/skin color declared	98.2	78.6
2. Schooling level	33.9	37.2
3. ZIP Code^e^ of place of residence	95.9	100.0
4. Geographic area of residential address	96.6	88.6
5. Case arising from flu-like syndrome outbreak	93.5	71.0
6. Case of SARS with hospital-acquired infection	92.5	69.9
7. Case with direct contact with birds, pigs, others	90.7	66.1

a) SARS: Svere acute respiratory syndrome; b) SIVEP-Gripe: Influenza Epidemiological Surveillance Information System; c) Hospitalized SARS cases living in the municipalities belonging to the Regional Center of Ubá, state of Minas Gerais, Brazil; d) SARS cases notified in the national territory; e) ZIP Code: Postal Address Code.

On the national database, it could be seen a lower percentage of completeness of the variable 'geographic area of the residential address', classified as 'regular' (88.6%), and 'self-declared race/skin color', classified as 'poor' (78.6%), while the same variables presented completeness greater than 95%, classified as excellent level, on the regional database. The variable 'case with direct contact with birds, pigs, others' presented a level of completeness classified as 'good' on regional database, while it was classified as 'poor' at the national level.

Table 2 shows the percentage of completeness of the variables related to the clinical condition and evolution of the case. Of the 49 variables evaluated, it could be seen that the completeness of 'excellent' filling occurred in 13 on the regional database, while on the national database, it occurred in only eight. The variables 'date of discharge from the ICU' and 'date of discharge or death' presented a level of completeness classified as 'very poor' (19.9% and 46.6% respectively) at the regional level, and 'poor' or 'regular' (51.8% and 89.9% respectively) at the national level.

**Table 2:** Completeness of the blocks of variables related to clinical condition and evolution of hospitalized SARS^a^ cases (n = 1,192,518), at the regional and national levels, according to SIVEP-Gripe,^b^ for the Regional Center of Ubá/MG and Brazil, 2020

Variables	Completeness	
	Regional^c^ (%)	National^d^ (%)
	(n = 2,590)	(n = 1,192,518)
**Signs and symptoms**		
1. Fever	96.6	85.3
2. Cough	97.4	87.6
3. Sore throat	94.8	72.6
4. Dyspnea	97.7	87.7
5. Respiratory distress	96.9	82.2
6. Saturation < 95%	96.1	82.2
7. Diarrhea	94.7	71.7
8. Vomiting	94.4	70.6
9. Abdominal pain	91.5	42.0
10. Fatigue	91.3	43.2
11. Loss of smell	91.2	41.6
12. Loss of taste	91.1	41.9
13. Other signs and symptoms	90.1	71.1
**Risk factors**		
14. Presents a risk factor	100.0	100.0
15. Puerperal woman	61.6	39.1
16. Chronic cardiovascular disease	62.7	51.1
17. Chronic hematologic disease	61.2	39.3
18. Down syndrome	61.3	39.3
19. Chronic liver disease	61.2	39.2
20. Asthma	61.4	40.2
21. Diabetes *mellitus*	62.7	47.5
22. Neurological disorders	61.5	40.5
23. Chronic lung disease	61.6	40.5
24. Immunodeficiency ou immunodepression	61.2	39.8
25. Chronic kidney disease	61.5	40.2
26. Obesity	61.3	39.8
26.1 Body mass index - BMI (obese)	19.1	47.3
27. Other risk factors	60.7	46.8
27.1 Description of other morbidities	98.0	98.8
**Vaccination history**		
28. Got vaccinated against influenza in the last campaign	72.3	40.1
28.1 Date of his/her last dose of influenza vaccine	66.9	66.7
29. His/her mother got vaccinated against influenza (for those under 6 months old)	47.8	29.8
30. Mother breastfeeds her child (for those under 6 months old)	39.1	30.1
**Other clinical information**		
31. Used antiviral medications to treat the disease	83.6	72.3
31.1 Antiviral medication used (oseltamivir, zanamivir, other)	94.7	93.8
31.2 Description of another antiviral medication used	88.9	70.8
31.3 Date of antiviral treatment initiation	93.1	91.9
32. Need for hospitalization	98.8	97.0
32.1 Date of hospitalization	96.2	98.3
32.2 Federative Unit where the hospitalization occurred	95.4	100.0
32.3 Municipality where the hospitalization occurred	95.4	100.0
32.4 Sentinel unit where the hospitalization occurred	95.4	100.0
33. Need to be hospitalized in the ICU^e^	92.2	83.5
33.1 Date of admission in the ICU	99.0	97.7
33.2 Date of discharge from the ICU	19.9	51.8
34. He/she used ventilatory support	86.9	82.1
35. Evolution of the case	87.7	85.5
35.1 Date of discharge of death	46.6	89.9
36. Date of case closure	88.0	88.2

a) SARS: Severe acute respiratory syndrome; b) SIVEP-Gripe: Influenza Epidemiological Surveillance Information System; c) Hospitalized SARS cases living in the municipalities belonging to the Regional Center of Ubá, state of Minas Gerais, Brazil; d) SARS cases notified in the national territory; e) ICU: Intensive care unit.

Regarding the block of variables related to signs and symptoms, it could be seen that, at the national level, these variables presented 'regular' completeness for fever, cough, dyspnea, SpO_2_ < 95%, 'poor' completeness for sore throat, diarrhea and vomiting, and 'very poor' completeness for abdominal pain, fatigue, loss of smell and loss of taste. At the regional level, the level of completeness was 'excellent' for fever, cough, dyspnea, SpO_2_< 95%, and 'good' for the other variables related to signs and symptoms.

**Table 3:** Completeness of the blocks of variables related to diagnostic criteria of reported hospitalized SARS^a^ cases (n = 1,192,518), at the regional and national levels, according to the SIVEP-Gripe,^b^ for the Regional Center of Ubá/MG and Brazil, 2020

Variables	Completeness	
	Regional^c^ (%)	National^d^ (%)
	(n = 2,590)	(n = 1,192,518)
**Imaging tests**		
1. Chest X-ray	67.6	55.7
1.1 Specifications of other chest X-ray results	63.0	86.6
1.2 Date of chest X-ray	87.0	90.6
2. CT scan aspect	35.7	32.4
2.1 Date of CT scan procedure	88.1	90.3
**Sample collection**		
3. Sample collection was performed for diagnostic test	98.3	96.3
3.1 Date of sample collection for diagnostic test	100.0	100.0
3.2 Type of clinical sample collected for diagnostic test	98.8	97.9
3.3 Specification of another sample that was collected	98.5	96.0
**Antigen rapid test**		
3.4 Type of antigen rapid test that was performed	34.8	41.0
3.4.1 Date of antigen rapid test result	9.2	6.9
3.5 Antigen rapid test result	94.3	84.8
3.5.1 Positive antigen rapid test result for influenza	2.2	2.9
**RT-PCR^e^**		
3.6 RT-PCR test result/another method applying molecular biology	98.3	99.2
3.6.1 Date of RT-PCR test result/another method applying molecular biology	92.4	89.8
3.7 Positive RT-PCR test result for influenza	93.8	21.8
3.7.1 Positive RT-PCR test result for influenza A or B	100.0	99.8
**Serological test**		
3.8 Type of serological sample that was collected	4.8	8.5
3.8.1 Description of the type of clinical sample different from those afore mentioned	52.4	101.9^i^
3.9 Date of collection of the material for serological diagnosis	5.4	8.0
3.10 Type of serological test that was performed	3.5	8.9
3.10.1 Description of the type of serological test that was performed	100.0	0.2
3.11 Serology result for SARS-CoV-2 IgG^f^	4.0	10.8
3.12 Serology result for SARS-CoV-2 IgM^g^	4.1	11.1
3.13 Serology result for SARS-CoV-2 IgA^h^	0.8	6.8
**Outcome**		
4. Final classification of the case	94.0	93.9
4.2 Indicate confirmation criterion	92.8	91.4

a) SARS: Severe acute respiratory syndrome; b) SIVEP-Gripe: Influenza Epidemiological Surveillance Information System; c) Hospitalized SARS cases living in the municipalities belonging to the Regional Center of Ubá, state of Minas Gerais; d) SARS cases notified in the national territory; e) RT-PCR: Reverse transcriptase polymerase chain reaction; f) Individuals with antibodies to SARS-CoV-2 by immunoglobulins G; g) Individuals with antibodies to SARS-CoV-2 by immunoglobulins M; h) Patients with antibodies to SARS-CoV-2 by immunoglobulins A; i) Classification that was not performed due to inconsistency.

Table 3 presents the result of the analysis of the completeness of variables related to the diagnostic criteria. Of the 27 variables in this block, only six presented an 'excellent' classification, both on the regional and national databases. It could be seen that this block presented the highest number of variables whose completeness level was classified as 'very poor', both on the regional and national databases.

## Discussion

Based on the analysis performed, it could be seen that the completeness of the notifications of hospitalized SARS cases, reported on SIVEP-Gripe, was higher on the RHC/Ubá than on the national database, taking into consideration the highest frequency of variables with level of completeness classified as 'excellent' or 'good' on this regional database.

The lowest completeness presented on the national database may result from disparities in access to information among the different Brazilian macro-regions. The structure and quality of health information systems in Brazil are not homogeneous, and there are many locations where paper notification forms are used to fill in data that need to be digitalyzed.^8^ It is worth mentioning that the health region included in this study enter data manually into the health system, that is, the paper forms after being filled out are sent to the municipal epidemiology services in order for them to enter the data into SIVEP-Gripe.

Regarding the variables related to sociodemographic characterization of the reported cases, it is worth highlighting the low completeness of the variable 'schooling level', classified as 'very poor' both on the regional and national databases. Despite the importance of information on schooling level for planning social and health policies, it can be seen that these data have been neglected in different information systems, especially in the Notifiable Health Conditions Information System (Sinan) and Mortality Information System (SIM).^14^ ^,^ ^16^

On the national database, we observed that the variable 'self-declared race/skin color' showed poor completeness, possibly attributable to the individual perception regarding their race/skin color.^15^ Such data can provide information about the living and health conditions of a given population, especially in a pandemic context with numerous gaps related to epidemiological aspects of COVID-19.^17^

With regard to the variables 'case arising from flu-like syndrome outbreak', completeness was 'poor' in the national context and satisfactory in the regional context. This information gap makes it difficult health surveillance actions in the territory,^18^ given that, in the face of flu-like syndrome outbreaks, public services seek combinations of contextual factors and public/private actions to reduce and mitigate the transmission of COVID-19 during the critical initial stage. This information contributes to the implementation of preventive and interventional measures aimed at reducing viral circulation in order to prevent the spread of the disease and the occurrence of subsequent cases arising from community transmission.^7^

The level of completeness of the variable 'case with direct contact with birds, pigs, others' was classified as 'good' on the regional database, although it was classified as 'poor' on the national database. COVID-19, responsible for most hospitalizations for SARS in 2020, is a zoonotic disease. Therefore, it is worth highlighting the importance of the adequate completeness of the national data of this variable, as information about direct contact between people and the disease and animals.^2^ ^,^ ^12^ ^,^ ^20^

When analyzing the block of variables related to clinical manifestations reported on SIVEP-Gripe, it could be seen that signs and symptoms such as sore throat, diarrhea, vomiting, abdominal pain, loss of smell and loss of taste presented level of completeness classified as 'poor' in the national database, but on the other hand, 'good' within the regional health. Inconsistent information on clinical data of notified cases constitutes a limitation for its closure, given that these data are based on laboratory, imaging, epidemiological and clinical criteria.^2^ ^,^ ^21^ ^,^ ^23^

Regarding the variables related to influenza vaccination history, 'poor' and 'very poor' levels of completeness were observed in the regional and national databases. It is worth highlighting a study conducted in Brazil, in which found better outcomes among people who caught COVID-19 shortly after getting vaccinated against influenza.^24^ ^,^ ^25^ Thus, the low completeness of this variable in the databases may limit the performance of this analysis. Another variable that showed low completeness was that related to the use of antiviral drugs, whose level of completeness was classified as 'poor' on the national database and 'regular' on the regional database, although it is recommended in the influenza treatment protocol in patients hospitalized for SARS.^1^ ^,^ ^12^

There was low completeness of the variables related to ICU admission, such as the date of ICU discharge and the date of discharge or death, classified as 'very poor' on the regional database and poor' (date of ICU discharge) or 'regular' (date of discharge or death) on the national database. The low completeness of the variables related to the occupation of ICU beds compromises the calculation of the occupancy rates of beds intended for SARS cases and thus makes it impossible to make a more accurate assessment of the real situation.^15^ ^,^ ^18^ It is believed that the expressive number of variables with 'poor' level of completeness is a consequence of the lack of motivation or insufficient time to complete the forms, given the prioritization of urgent demand for health services in the context of the pandemic.^26^ ^,^ ^27^

The variables related to data on imaging tests for diagnostic confirmation, although considered important in an epidemiological investigation, also presented low completeness, both at the national and regional levels.^6^ ^,^ ^29^ With regard to laboratory tests, the variable 'Was a sample collected from you for diagnostic test?' was classified as 'excellent' in both databases. If, according to current protocols, it is recommended that all symptomatic individuals should take RT-PCR or antigen rapid test, these tests were not performed for all hospitalized SARS cases. This result may reflect the challenges facing the national scenario, marked by insufficient financial, human and material resources, obstacles to the performance of extensive testing.^12^ ^,^ ^19^ ^,^ ^30^

The variable 'final classification of the case' presented a level of completeness defined as 'good' on the regional and national databases, although it should be 'excellent' because this variable related to the final diagnosis of the case.^27^ Thus, there is a limitation of SIVEP-Gripe as to the accuracy of this information, given that in such cases the case closure was carried out without the necessary information for its final classification having been duly recorded. These gaps show failures in the follow-up of laboratory test results, as well as in the return flow to the system for the inclusion of the result and case closure.^26^Thus, we suggest a possible change in operational standardization, reclassifying the fields considered 'essential', such as tests and final classification of the case, as 'mandatory', which would improve the completeness of these variables.^15^ ^,^ ^21^

Basically, when analyzing the completeness of filling the SIVEP-Gripe on a database at regional and national levels, we identified low completeness of the variables, which can make it difficult the definition of the epidemiological profile of cases and, consequently, surveillance actions. As strengths, this research shows results of the analysis of the completeness of filling the SIVEP-Gripe, a health information system that has been frequently updated during this COVID-19 pandemic. Our findings may support actions aimed at improving the adequate filling of the SIVEP-Gripe, especially if changes are made to the operationalization of variables whose completeness was classified as 'poor' or 'very poor'. Such changes should be planned in order to make it more sensitive, active and able to monitor the event in the territory, in a timely and efficient manner.

The limitation of the study is related to the impossibility of removing duplicates from the national database, due to the lack of access to individual data, which may have affected the classification of completeness of some variables.

Taking these results, it could be seen that during the COVID-19 pandemic in 2020, both databases, evaluated in this study, presented low completeness of hospitalized SARS case notifications. The lack of information at the national level was even greater, reflected in the greatest number of variables presenting the worst completeness. It is recommended that epidemiological surveillance services develop routine periodic evaluation of the most critical variables, regarding low filling, aimed to eliminate low completeness of reported data and its commitment to the quality of SIVEP-Gripe. Finally, it is suggested improvements in the work process, through updates and routine training of professionals responsible for filling out notification forms and management of the Influenza Epidemiological Surveillance Information System.
